# Development of a novel observed structured clinical exam to assess clinical ultrasound proficiency in undergraduate medical education

**DOI:** 10.1186/s13089-023-00337-2

**Published:** 2023-09-25

**Authors:** Andrew Kamilaris, Jeffrey A. Kramer, Gwen Baraniecki-Zwil, Frances Shofer, Christy Moore, Nova Panebianco, Wilma Chan

**Affiliations:** 1 Department of Emergency Medicine, Yale University, New Haven, CT USA; 2 Department of Emergency Medicine, University of Pennsylvania, Philadelphia, PA USA

**Keywords:** Medical education, Medical students, Clinical ultrasound, Point of care ultrasonography, Undergraduate medical education

## Abstract

**Objectives:**

A pilot study was performed to develop and test an observed structured clinical exam (OSCE) for clinical ultrasound in second-year medical students. The goal was to assess a longitudinal clinical ultrasound curriculum for medical students and to help determine readiness to perform ultrasound during clinical clerkships.

**Methods:**

The OSCE contained 40 tasks over 30 min in a one-to-one examiner to examinee environment using standardized patients covering cardiac, pulmonary, and inferior vena cava (IVC) ultrasound exams along with 6 critical diagnoses. Examinees were assessed using a binary checklist approach. A two-way ANOVA analysis was performed to determine if there were differences between the day and session the OSCE was administered. Results are presented as mean ± standard deviation.

**Results:**

One hundred fifty-two students were tested with an overall mean score of 64.9 ± 17.6%. Scores between the cardiac, IVC, and lung sections varied—67.8% ± 18.8%, 62.4% ± 26.2%, and 57.1% ± 20.6%, respectively. One hundred twenty-six (82.9%) answered at least one critical diagnosis incorrectly. Students in the late session performed better than the early session (1: 60% vs 2: 69%, *p* = .001).

**Conclusions:**

Students performed better in later sessions. Additionally, the number of questions left blank at the end of the exam suggests that the length of the OSCE should be evaluated. Incorporating critical diagnoses was challenging for examinees. The proposed OSCE is a valuable assessment tool that could be adapted to assess student’s readiness to use clinical ultrasound prior to clerkships.

## Introduction

Clinical ultrasound (CUS) allows for physician-performed, rapid bedside evaluation, and interventional management with portable ultrasound equipment [2]. Traditional comprehensive or radiology performed ultrasonography requires more time and resources, which may include sonographic technicians, radiologists, patient transporters, and radiology suites. This increases time to intervention and ultimately patient disposition, which further strains medical systems. CUS is not meant to replace comprehensive radiology studies. It aids in making informed clinical decisions at the bedside quicker and improves procedural safety [15, 19].

CUS has shown to decrease time to an accurate final diagnosis and decrease resource utilization [10]. This is also true for learners who use CUS, showing that their use of ultrasound improved their diagnostic performance significantly during simulated cardiorespiratory scenarios [17]. CUS integration within undergraduate medical education (UME) in anatomy and physiology, physical examination, pathology, and procedural skills modules has shown to improve knowledge, understanding, confidence, and learner satisfaction [7, 8, 12, 15, 17, 21]. While many medical schools have developed longitudinal CUS curricula, there is heterogeneity in scope, content, and adoption [1, 2, 7, 14].

CUS educators have adapted to the changing landscape of medical education and demonstrated that teaching can be successful in various formats including flipped-classroom models, asynchronous learning, web-based modules, videos, and simulation in addition to traditional classroom-based or clerkship learning [12, 14, 21]. While UME CUS education was historically taught using more expensive, higher image quality cart-based machines with limited availability, the rapid improvement in image quality, affordability, and attainability of ultra-portable handheld ultrasound devices has expanded CUS availability to students [9, 16, 20, 22]. Educational standards for UME CUS have been proposed, but not yet widely adopted [1, 2, 13]. With greater accessibility necessitates the need for enhanced governance, accountability, and oversight.

CUS proficiency can be divided into three pillars: image acquisition, image interpretation, and integration of the interpretation into medical decision making [12]. The Accreditation Council for Graduate Medical Education (ACGME) and the American College of Emergency Physicians (ACEP) have developed criteria to assess CUS proficiency for residents in emergency medicine, but in the UME realm this is largely left up to individual institutions to determine [11]. An observed structured clinical examination (OSCE) is an educational tool used to assess whether a student has obtained the clinical skills and expectations defined by an educational curriculum [3, 4]. OSCEs have been widely utilized and endorsed by major national organizations because of their utility with novice learners, repeatability, and realistic patient care scenarios [18].

In this manuscript, we describe the development and implementation of a UME OSCE that is based on the principles defined by Harden [5] and Hodges [6]. The pilot OSCE evaluates the three levels of mastery (acquisition, interpretation, and medical decision making) in predefined areas of core ultrasound knowledge applicable across multiple medical specialties: cardiac, pulmonary, and inferior vena cava assessment [12].

The purpose of this study was to assess the feasibility of using the OSCE tool during medical school training and determine student CUS proficiency prior to entering clinical rotations.

## Methods

### Study design, setting and population

This is a descriptive study of a novel UME CUS OSCE using a binary checklist approach as previously described by Hoppmann et al. [7] and adapted to meet our goals. The OSCE was implemented as part of the ultrasound curriculum for second-year medical students at an academic medical center during their “bootcamp” prior to clerkships in December 2021. The study was exempt from the Institutional Review Board (IRB). The pilot OSCE was formative and did not go on the student’s academic record.

The study population included second-year medical students at the end of their third semester of pre-clerkship training. These students have already received three semesters of flipped-classroom style ultrasound curriculum incorporated into a pass–fail course during their preclinical curriculum. The flipped-classroom ultrasound curriculum included small group instruction and hands-on training sessions that were interspersed during their preclinical training. All students who participated in the course passed and there was no objective assessment as there were no prior OSCEs in the curriculum prior to this pilot. Students were informed of the OSCE topics being assessed a week prior via email. No additional details or checklists were provided.

### OSCE examination content and administration

The OSCE contained three main modules: cardiac, pulmonary, and inferior vena cava. These modules were chosen as core essentials for all students, regardless of future specialty. Students had 30 min to complete 40 tasks consisting of professionalism, proper introductions and hand washing, technical and diagnostic sonography skills such as imaging indication, probe selection, image acquisition, interpretation, pathology identification, troubleshooting techniques, and imaging ergonomics were evaluated. Within the pathology identification section there were six critical diagnoses including pericardial effusion, right heart strain, decreased ejection fraction, pneumothorax, b-lines, and pleural effusion. To reduce content leak and to preserve time, feedback was withheld during this pilot OSCE.

The OSCE was conducted on standardized patients (SPs). Video clips displayed on tablets or laptops were interspersed throughout the exam to display pathological images for some of the checklist items. The exam was administered with the assistance of ultrasound faculty, fellows, and trained ultrasound teacher’s assistants (TAs). TAs are fourth year medical students with a particular interest in ultrasound that help teach throughout the year. The TA program was started in 2019 prior to this pilot and have helped with small group instruction in the pre-existing ultrasound curriculum. All TAs have already completed the classroom portion of the already existing ultrasound curriculum, a 60-min TA training session at the beginning of the academic year, and a 30-min training session on OSCE administration. They were also given an examiner guide that described criteria for grading which was also available during the exam.

Students in the preclinical bootcamp were divided into two sessions, early and late, over 4 days to accommodate the number of participants. Students examined in the later session participated in ultrasound image review and didactics prior to their OSCEs; earlier examinees participated in ultrasound image review and didactics after completing their OSCEs. The OSCE session was a scheduled one-on-one examiner to examinee encounter. A minimum of five TAs and five SPs were required to administer the exam.

### Measurements and data analysis

Examiners tabulated student scores on paper copies of the 40-item OSCE checklist. Each item was graded on a binary scale and received a ‘1’ or ‘0’ for each task depending on if they completed it successfully or not. The critical diagnoses were deemed an essential part of the exam, and the student would ‘fail’ if they did not successfully identify the diagnosis. This is like critical actions in the American Board of Emergency Medicine (ABEM) oral board exam. The checklist is included here for reference: https://tinyurl.com/bdcvcevw.

Total percent correct was calculated in two ways: (1) blank responses coded as 0, where the denominator was all tasks (*n* = 40) and (2) blank responses were coded as missing, where the denominator included only tasks attempted. Additionally, scores were calculated for the three sub-sections (cardiac, IVC, and lung) in the same manner as described above. To determine differences in scores between session (first or second) and session day (*n* = 4), a two-factor analysis of variance (ANOVA) was performed. To determine differences in scoring by examiner or differences between sub-sections (cardiac, IVC, lung), a one-way ANOVA was performed. To adjust for multiple comparisons, post-hoc pairwise Tukey–Kramer tests were used. Paired *t* tests were used to assess differences in scoring with and without blanks. Summary statistics are presented as means with 95% confidence intervals unless otherwise noted. All analyses were performed using SAS statistical software (version 9.4, SAS Institute, Cary NC).

## Results

### General

Out of the possible 160 students eligible to participate in the OSCE, 152 students participated. Twelve examiners administered the OSCEs which varied by day and session. There were between 16 and 24 students and 5–7 examiners per session (Table 1).

**Table 1:** Number of students per session and examiners present in each session

Day	Session	*N* (%)	Examiners present
1	Early	20 (13)	5
	Late	19 (13)	7
2	Early	18 (12)	5
	Late	24 (16)	7
3	Early	16 (11)	6
	Late	20 (13)	7
4	Early	16 (11)	6
	Late	19 (13)	5

### ***Students’ performance on the OSCE***

**Fig. 1:**
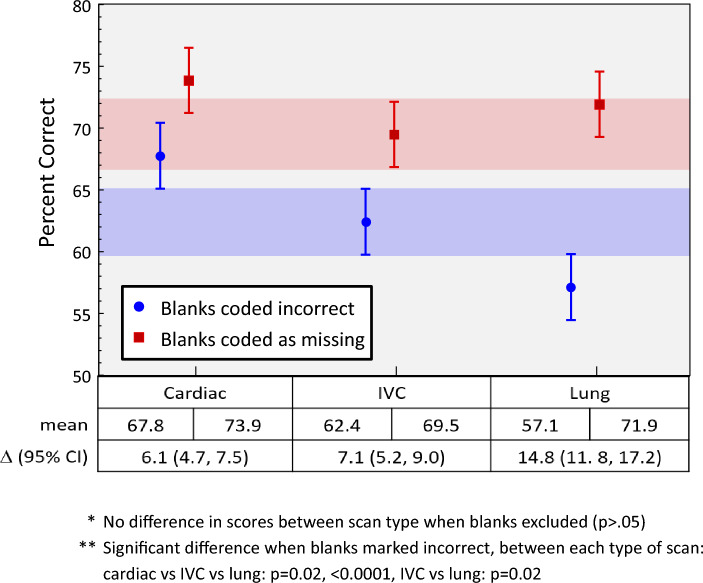
Scores by subsection

The overall mean OSCE score when blanks were coded as incorrect was 64.9%. Overall means scores were significantly higher when missing was excluded (74.6%, difference = 9.7%, 95% CI 8.2, 11.2%, *p* < 0.0001). When scores were examined by subsection, students performed significantly better in the cardiac section (67.8%) followed by IVC (62.4%) and lung (57.1%), all post-hoc pairwise comparisons statistically significant, *p* < 0.02 (Fig. 1). When unanswered questions were removed, there was no significant difference between the section scores (74%, vs 70%, vs 72%, *p* > 0.05 for all post-hoc pairwise comparisons, Fig. 1).

**Fig. 2:**
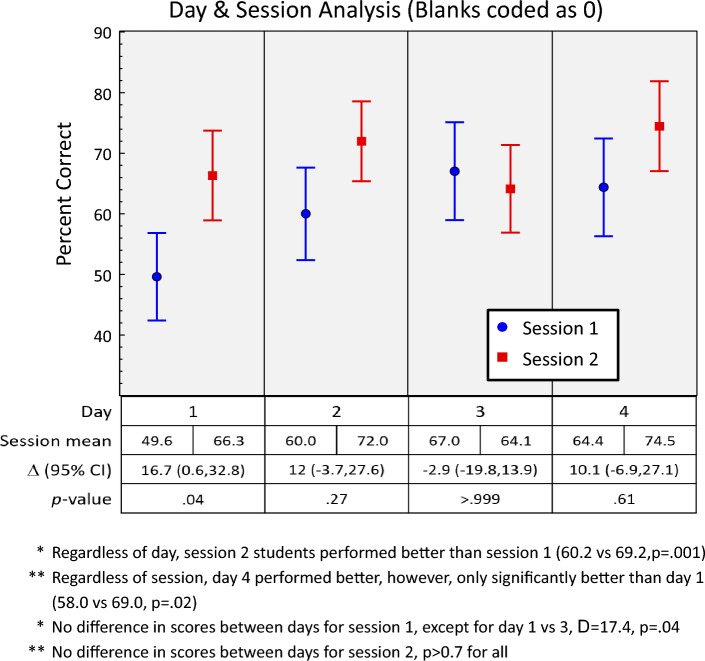
Scores by day and session

Overall, the session 2 (late morning) performed significantly better than session 1 (early morning) (69% vs 60%, difference = 9.0 95% CI 3.7%, 14.2%, *p* = 0.001, Fig. 2). Additionally, regardless of session, day 4 of bootcamp students performed better compared to all other days (58%, 66%, 66%, 69% for days 1–4, respectively); however, only day 1 and 4 were significantly different (*p* = 0.02).

**Fig. 3:**
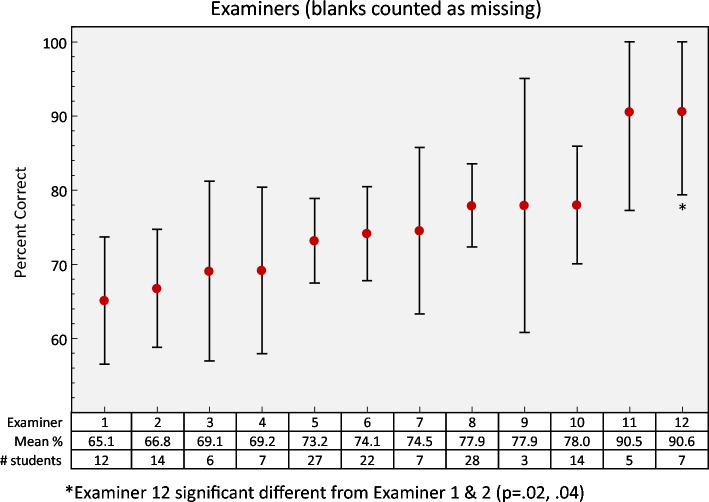
Scores by examiner. Examiners 2 and 5 are faculty

Examiner mean scoring ranged from 65.1 to 90.6%, with 50% of the mean scores between 69.0 and 78.0% (Fig. 3). Both examiners 11 and 12, whose mean scores were > 90%, were examiners on the last day and session of bootcamp.

**Fig. 4:**
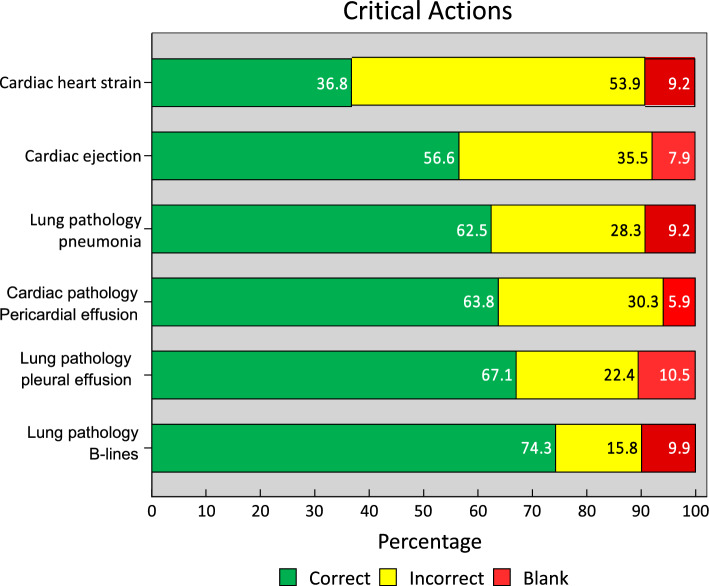
Scores of critical actions

One hundred twenty-six students (83%) answered at least one critical diagnosis incorrectly. B-lines was the critical action most interpreted correctly (74.3%) whereas identification of right strain was the least (36.8%) (Fig. 4).

## Discussion

### Logistics discussion

Ultimately, the OSCE for clinical ultrasound was able to be integrated into a bootcamp for medical students about to start their clinical clerkships. Although our OSCE is not comprehensive, it assesses what we believe to be core uses for clinical ultrasound, regardless of specialty. A major strength of the OSCE was the breadth of skills assessed. Compared to previous ultrasound OSCEs, this approach of evaluating a broad set of skills is novel. Our OSCE assessed professionalism, including introductions and hand washing, as well as technical and diagnostic sonography skills such as imaging indication, probe selection, imaging mechanics and ergonomics, image acquisition, interpretation, pathology identification, and troubleshooting techniques. We believe that adding these components can better assess a sonographer’s skill set, and that image acquisition should not be the sole item evaluated.

It should be noted that this examination was administered at a major urban academic medical school that is well resourced. Our ultrasound TA program, availability of SP’s, ultrasound faculty, fellowship program, and dedicated teaching sonographer all contributed to this effort. Obviously, not all schools will have these resources readily available, and this could be a potential barrier to implementation. The budget to administer the exam included the time for the SPs. There were no other expenses incurred.

### Scoring and results discussion

The scoring of the OSCE is an important topic of discussion and can play a role in how to use the results of this pilot and exam. The binary approach to scoring was used to help decrease subjectivity, and allows for fast, straight-forward scoring. The incorporation of critical diagnoses was added to ensure that students can acquire and interpret key pathology that would require intervention. It is essential that students recognize crucial pathology to avoid misdiagnoses. This is a vital point given that clinical ultrasound is not something all supervising physicians are facile with and may rely on a trainee’s interpretation of the result. Supervising physicians unfamiliar with ultrasound should not take this approach. Providers should use this new tool in accordance with their comfort level and obtain a comprehensive or radiology ultrasound when needed.

The TA program and training session were major strengths that strived to decrease subjectivity in the scoring of the OSCE. The binary checklist scoring system helped decrease variability in scoring among examiners as seen in the results, however, we did not assess inter-rater reliability (Fig. 3). Additionally, all the TA’s are well-versed in clinical ultrasound and participated in a training session on how to administer the OSCE and had an examiner guide on scoring to reference. A factor that likely influenced scores and could be modified was the timing of the OSCE compared to the didactic session. Not surprisingly, those who had didactics prior to the OSCE exam had an advantage. Although this was unavoidable during our initial pilot, this could be modified in future implementations of the exam. Similarly, exam performance improved as the week went on. This was likely due to student discussion of the exam, which was discouraged but likely still occurred.

Many students did not finish the exam, and most of the blank responses were in the lung section, which was the last section of the exam. Future iterations of the exam could randomize question order to determine whether the lung section was left blank because of difficulty or timing. Although examinees had the opportunity to skip questions and come back to them, it is unclear whether the number of blank responses indicate there was not enough time for the OSCE or that the OSCE was too long, too difficult, or a combination of these. 83.0% of students missed at least one critical diagnosis. The longitudinal curriculum in ultrasound covers many topics. Along with all of their other coursework, ultrasound may not be a top priority for students, or they may not have had significant exposure to these critical diagnoses. In addition, the assessment was formative and not graded, but it seems that many students got stuck trying to acquire one view or answering a particular question instead of skipping and moving on. This could indicate that the students were unfamiliar with this type of assessment where timing is of the essence or were unsure of the contents of the OSCE. A checklist of tested items could be provided in advance for future OSCE administrations.

We propose a scoring system with a minimum passing score, such as 75% (30 out of 40 checklist items). Students must also identify each critical diagnosis item to pass. Importantly, the students will receive a dedicated one-on-one feedback session at the end of each OSCE to identify strengths and weaknesses and develop ways to improve. Ideally, students will have these clinical ultrasound OSCEs integrated throughout their preclinical curriculum and can be used as a springboard for other related lessons [3].

The OSCEs in the preclinical curriculum would be formative OSCEs (FOSCE), which are not formally scored, but are an opportunity for students to hone their skills and receive feedback. The OSCE will be formally scored with the proposed scoring regimen right before their clinical clerkships begin. This will be known as the summative OSCE (SOSCE). Although the student performance on this pilot OSCE was lower than expected, it is likely that with the above curriculum almost all students would ‘pass.’ It is proposed that the students will be allowed to use their handheld device during their clerkships only after receiving a passing score on their summative OSCE. Given the plethora of opportunities during the preclinical years to take FOSCEs and improve their skills, it is unlikely any students would fail. This will serve as a mechanism to ensure students have the skills and knowledge to use clinical ultrasound safely.

### Future directions and conclusion

Ultimately, this pilot study was a success. We were able to successfully develop and implement a novel OSCE incorporating our desired metrics, which met the main goal of the study. Of course, there were limitations as previously discussed and there is much work to be done in this area with room for improvement and expansion. The creation of a more generalizable exam that could be administered in medical schools with less resources is an example. Ideally, formative OSCEs will be integrated seamlessly within the preclinical curriculum and into sessions involving anatomy, problem-based learning, pathology, and physical exam skills. This will require support from medical school deans and administration.

Future directions include studying the reliability and validity of the assessment. Another goal could be to conduct the proposed OSCE at other institutions to create a more standardized assessment. Major barriers to this type of assessment are time and resources. Technology is rapidly evolving and utilizing virtual reality and/or augmented reality simulators along with artificial intelligence may help reduce faculty burden and aid in OSCE administration. This may allow for more frequent and formative OSCE administration.

In summary, there is increasing inclusion of CUS in UME curricula; however, proficiency assessment tools are limited. OSCEs have repeatedly shown to be a valuable assessment tool in medicine, and our OSCE exam is one that could be adapted to assess students' readiness to use clinical ultrasound prior to clinical clerkships. Our proposed system of repeated formative assessment with a final summative assessment will help students assess their CUS strengths and weaknesses to continuously strive for improvement of their knowledge, skills, and confidence to start exploring the field of clinical ultrasound.

## Data Availability

The data sets used during the current study are available from the corresponding author on reasonable request.

## Abbreviations

CUS: Clinical ultrasound
UME: Undergraduate medical education
ACGME: Accreditation Council for Graduate Medical Education
ACEP: American College of Emergency Physicians
OSCE: Observed structured clinical exam
IRB: Institutional Review Board
SP: Standardized patient
TA: Teacher’s assistant
ABEM: American Board of Emergency Medicine
ANOVA: Analysis of variance
